# ‘I don’t want to be a guinea pig’ - Swedish women’s experiences of breast abscess treatment

**DOI:** 10.1186/s12905-024-02937-z

**Published:** 2024-02-08

**Authors:** Margareta Johansson, Lisa H. Amir

**Affiliations:** 1 Department of Women’s and Children’s Health, Uppsala University, Akademiska University Hospital, SE-751 85 Uppsala, Sweden; 2 Judith Lumley Centre, School of Nursing & Midwifery, La Trobe University, Bundoora, Victoria Australia; 3 Breastfeeding Service, Royal Women’s Hospital, Melbourne, Australia

**Keywords:** Experiences, Interview, Puerperal breast abscesses treatment, Women

## Abstract

**Background:**

It is well known that breastfeeding plays an important role in the health of women and children. However, women are not always given optimal support and most do not reach their breastfeeding goals. About one in five, breastfeeding women report mastitis and a small proportion of these develop a breast abscess. Our aim was to describe the experiences of a group of Swedish breastfeeding women who developed a breast abscess.

**Methods:**

A qualitative cross-sectional study with 18 study participants was undertaken in Sweden in 2017–2018. Potential participants were identified through electronic medical records at a university hospital and invited to participate in audio-recorded telephone interviews. Women were between 2 and 24 months postpartum at the time of the interview, on average 8 months. We conducted a thematic analysis in six steps according to Braun and Clark.

**Results:**

Our analysis identified two themes: 1) Seeking care and receiving treatment was long and unpleasant, and 2) Importance of adequate professional care. Women who experienced a breast abscess were uncertain about where to ask for professional help. They often had a long wait for the right time to undergo the unpleasant and painful procedure of draining their breast abscess. The women felt it was important to receive professional care with respectful communication, continuity of care, and to receive adequate information, but they did not always receive this level of care.

**Conclusions:**

Women with puerperal breast abscesses often fall between medical specialty areas. No longer under the care of obstetricians and maternity services, their problem is too complicated for general practitioners or emergency departments, but not regarded as serious by breast surgeons. Healthcare professionals urgently need adequate training in order to deal with breastfeeding problems and be able to offer women-centred care.

**Supplementary Information:**

The online version contains supplementary material available at 10.1186/s12905-024-02937-z.

## Background

It is well known that breastfeeding is important for the health of women and children [1]. However, women are not always given optimal support for breastfeeding and many do not reach their breastfeeding goals [2]. Breastfeeding problems, such as mastitis and breast abscesses, are often poorly managed by healthcare professionals, who lack training in the care of breastfeeding women [3–5]. In a recent interview study of women with mastitis in Norway: women felt they were not taken seriously by healthcare professionals [6]. To feel well supported as a patient, healthcare professionals need to be sensitive, pay attention, listen to and believe in women’s experiences [7]. Professional advice improves the rates of breastfeeding, but not all healthcare professionals have sufficient breastfeeding knowledge [8].

Mastitis means inflammation of the breast, and can be considered a continuum from non-infectious mastitis to infectious mastitis, which can develop into a breast abscess [9]. A puerperal breast abscess is defined as a collection of pus in the breast, with symptoms of pain and tenderness [10]. This is most common between 3 and 8 weeks of breastfeeding [11, 12], and experienced by less than 1% of women who commence breastfeeding [13, 14]. The development of an abscess can take time (days to weeks) as the area of inflammation becomes encapsulated and the core of the abscess liquifies [15].

*Staphylococcus aureus* is the most common organism isolated in the milk of lactating women with mastitis [12, 16, 17]; and breast abscesses are commonly treated with antibiotics, incision, and drainage or ultrasound-guided needle aspiration (See treatment options in Table 1) [18], but there is no consensus on the optimal treatment [19, 20]. Needle aspiration is the usual management and continued breastfeeding from the affected breast is recommended [21, 22]. Best results are achieved with aspiration using ultrasound guidance and with repeat aspirations performed as necessary until complete resolution [21]. Local anaesthetic is used for analgesia, and the addition of ice to the area may be beneficial [21]. Clinically unwell patients may need hospital admission for optimum care [23]. Trop et al. have proposed evidence-based algorithms for the diagnosis, management, and follow-up of breast abscesses [21]. These algorithms can be applied in the management of breast infections. Lactational breast abscesses are often challenging to manage and multidisciplinary management of breast abscess is required for optimal care.

**Table 1:** Treatment options for puerperal breast abscess [20]

Antibiotics	Antibiotics alone are insufficient in the management of breast abscess. There is insufficient evidence to determine whether antibiotics should be routinely prescribed in addition to drainage of collection.
Incision and drainage	The abscess is cut open with a scalpel to release the pus/fluid. Is typically performed when the abscess is multiloculated or has overlying early skin necrosis. A drain may be inserted into the wound to help the infected fluid drain or may be left open so that fluid drains naturally.
Needle aspiration	A needle is inserted into the cavity of the breast abscess and a syringe is used to draw out the infected fluid, often using ultrasound guidance. May need repeated aspiration. Less invasive than incision and drainage and is therefore preferred. Although in some cases incision and drainage is required if repeated aspiration fails.

While it is recommended to continue breastfeeding during and after puerperal abscess treatment, even if methicillin-resistant *Staphylococcus aureus* (MRSA) are isolated [22]; cessation of breastfeeding is not uncommon [24, 25]. Other complications related to puerperal breast abscess treatment include ongoing pain, mammary fistula, and unsightly scarring after open surgical drainage [6, 24].

Breastfeeding is complex with socio-economical, cultural, and psychosocial influences on women’s infant feeding methods [1]. Breastfeeding problems, such as a breast abscess, may have negative health, social and economic consequences for women, and prevent women reaching their breastfeeding goals [26].

### The Swedish breastfeeding support context

Services in Sweden for childbirth care and child health care are free of charge and reach almost 100% of families. During an uncomplicated childbirth, midwives are the primary caregivers, and if complications arise midwives co-operate with obstetricians [27]. Midwives support expectant parents to prepare for parenthood during antenatal care. The preparation includes information about benefits of breastfeeding, how parents can facilitate breastfeeding, and how to prevent breastfeeding complications [19]. The Swedish Child Health Service provides professional monitoring of children’s health, development and living conditions during the child’s first 5 years, by registered nurses and physicians [28].

In Sweden, exclusive breastfeeding at 1 week of age in 2021 was estimated to be 1.8 and 11.9% at 6 months [29]. Breastfeeding is facilitated by generous parental leave availability to mothers: 480 days (approximately 16 months) for one child [30].

When there are breastfeeding difficulties, additional support by midwives and physicians, which is available at 26 *Breastfeeding clinics* across Sweden. These clinics offer both parents and other healthcare professionals, advice and support over the telephone, video visits, and booked visits for breastfeeding issues [31]. Women in need of breastfeeding support either contact the clinics directly or are referred from outpatient care units [19]. The site of our study was a Swedish university hospital, which included one of these *Breastfeeding clinics*. Women treated for puerperal breast abscess at this hospital were offered follow-up appointments at the hospital’s *Breastfeeding clinic.* When other changes were identified in the breast, such as suspected breast cancer, the women were referred to the hospital’s *Breast center.*

A non-profit Swedish national breastfeeding association, The Swedish Nursing Mother’s Support [31], aims to promote breastfeeding, both through counselling and increasing knowledge, to parents as well as to healthcare professionals. The association was established in 1973 as a result of limited breastfeeding knowledge [32]. A free online breastfeeding support document in Swedish is available for the management of breast complications during breastfeeding [19].

Early diagnosis and management of inflammatory breast syndromes such as breast abscesses are important to reduce women’s suffering. There is limited understanding of breastfeeding women’s experiences of developing a breast abscess therefore, our aim was to describe the experiences of a group of Swedish women seeking diagnosis and treatment for a puerperal breast abscess.

## Methods

### Study design

Our approach to the research was a social constructivism worldview; the participants constructed the meaning of their varied and multiple experiences of breast abscess and a pattern of meaning was inductively developed [33].

### Participants and procedure

The inclusion criteria for participation in this qualitative cross-sectional study was women with treated puerperal breast abscess with sufficient knowledge of Swedish or English to conduct an interview. Exclusion criteria were not fully treated, untreated breast abscess, breast cancer or suspicion of cancer, men and non-puerperal breast abscess.

A retrospective review of electronical medical records at a university hospital in Stockholm, Sweden, was carried out to identify potential participants. The ICD-10 diagnostic codes for breast abscess (O91.1, O91.1A, O91.1B) [34], were screened in the medical records between 14 January 2017 and 7 September 2017, and 130 individuals identified.

Of the 130 women identified, 31 fulfilled the inclusion criteria and were invited to the interview study by the first author using a short text message with information about the study, between 18 September 2017 and 6 February 2018. Of the 31 potential participants, 13 did not answer the invitation and 18 agreed to participate and gave their written consent. Women were between 2 and 24 months postpartum at the time of the interview, on average 8 months. Thirteen of the 18 women regarded the time point the interview as preferable and five thought they had could not remember everything in detail. As a start of the interview, all participants gave oral consent to participate and were unknown to both authors prior to invitation to join the study. Following the consent process, these women were interviewed via telephone in a semi-structured interview between 19 September 2017 and 19 April 2018, at a time of their preference and audio-recorded. English was used in one interview and the others were conducted in Swedish. Interviews were transcribed verbatim in the language of the interviews by midwifery students. The first author closely supervised the transcription process, checked the accuracy by listening to the recorded transcripts, and discussed the content with the student transcribers. After coding, the first author selected representative quotes and these were translated into English, the transcripts were de-identified and assigned an interview number. The interview guide consisted of three overarching topics: *Please, describe your breast abscess experience*; *Please, describe your experience of the breast abscess treatment;* and *Please, describe your breastfeeding experience when treated for the breast abscess,* after an introductory explanation of the study. Follow-up questions were asked in order to get rich descriptions of women’s experiences. Background characteristics such as maternal age, parity, civil status, mode of the latest birth, and gestational age at that birth were also collected. The women talked freely without hesitation, and no one withdrew their participation. The timing of the interviews ranged from 30 to 87 minutes, with a mean duration of 56 minutes.

### Data analysis

Thematic analysis in six steps, according to Braun and Clark [35], was used for data analysis. (i) The first step was to be familiarized with the data by reading and re-reading the transcribed data and noting initial ideas that were discussed with the transcribers. (ii) The second phase involved generating initial codes with relevant features according to the aim, discussed with the second author in the end of this phase. (iii) The codes were then sorted into two potential themes: *Experiences before, during and after breast abscess treatment;* and *The importance of professional care*. (iv) Potential themes were reviewed by both authors and (v) finally, two main themes were describing breastfeeding women’s experiences of breast abscess (Table 2) [35]. The last step in the data analysis (vi) was to select vivid and compelling quotes in order to present the participants’ voices [35].

**Table 2:** The themes explored which were described by 10 sub-themes

Themes	Sub-themes
Seeking care and receiving treatment was long and unpleasant	Long waiting time and unsure where to receive help
	Assessment and diagnosis of the breast abscess
	Demanding experience of breast abscess when breastfeeding
	A long, protracted waiting time to undergo drainage of the breast abscess
	Mixed experience of the breast abscess treatment
	Treatment follow-up
Importance of adequate professional care	Continuity of care preference
	A wish for respectful communication and information sharing
	The importance of professionalism
	Role of caregiver’s gender in the care given

### Trustworthiness, rigour and reflexivity

The first author has several years’ experience as a clinical midwife, senior lecturer and researcher. The second author has worked in breastfeeding medicine for over 20 years in both general practice and a hospital setting, and many years’ experience as a researcher. Both authors have extensive experience in the care of women with mastitis and breast abscesses. During the process of designing the study, collecting and analysing data, critical discussions were carried out with the healthcare professionals at the hospital and at scientific conferences. At the end of each interview, participants were asked if the timing of the interview following their treatment for a breast abscess was appropriate. Most participants (*n* = 13) said it was an ideal time-point, four regarded it as appropriate but had forgotten some of their experience, and one considered that a long time had passed. However, all participants were willing to share their experiences in lengthy interviews. In the last interviews, women’s experiences started to repeat and no new information was obtained, so the decision was made to conclude the data collection. The Standards for Reporting Qualitative Research were used to ensure the trustworthiness and rigor of our study [36].

### Ethical considerations

The study was approved by the chief manager of the university hospital in Stockholm, and by the Ethical Review Board in Stockholm (Dnr. 2014/2265–31/4). All methods included in the study were performed in accordance with the relevant guidelines and regulations. All participants were given written and oral study information, informing them that participation was voluntary and that they were free to withdraw from participating without any explanation. Written and oral informed consent was obtained from all subjects Participants were identified in the transcriptions by an interview number and data were kept confidential.

## Results

### Description of the study participants

The 18 women included in this study were aged between 27 and 45 years (mean age 35 years), 16 (89%) lived with a partner, and 14 (78%) had given birth to their first child (Table 3).

**Table 3:** Sample characteristics

	Participants (*N* = 18)
	*n*
**Age** 27–45 years (mean = 35)	
**Parity**	
Primiparous	14
Multiparous	4
**Living with a partner**	
Yes	16
No	2
**Latest mode of birth**	
Spontaneous vaginal birth	11
Vacuum extraction	1
Elective caesarean delivery	1
Emergency caesarean delivery	5
**Gestational week at birth**	
Premature (< 37 + 0)	2
Term (≥37 + 0)	16

All participants had an experience of puerperal breast abscess that required treatment at hospital as an outpatient (Table 4). Only one participant had experienced a puerperal breast abscess in a previous lactation.

**Table 4:** Description of management for breast abscess

	Participants (*N* = 18)
	*n*
**Place of treatment**	
Breastfeeding university hospital centre	11
Different emergency units	7
**Management of absess**	
Pig-tail drainage only	8
Needle aspiration only	6
Needle aspiration and Pig-tail drainage (for two different abscesses)	1
Needle aspiration and drainage tube	1
Incision under general anaesthetic for placement of drainage tube	1
Rupture (one previous beast abscess)	1
**Antibiotics prescribed**	9

### Theme 1: seeking care and receiving treatment was long and unpleasant

The first theme ‘Seeking care and receiving treatment was long and unpleasant’ was described by six sub-themes, and can be seen in Table 2.

#### Long waiting time and unsure where to receive help

When the women suspected that a puerperal breast abscess had developed, they sought help from a range of services: the national telephone health care service, hospital emergency unit, hospital based breastfeeding centre, public health centre, public childcare centre and hospital-based postnatal care service. The women would have liked better information about where to turn for professional care and believed that it should have been easier to receive an appointment for breast abscess assessment by a physician.

Participants who attended an emergency department had a long waiting time before being seen. The women were not seen by a breastfeeding expert, they were questioned about why they had come and sometimes were even denied care. Two first-time mothers said:

*I felt a little stupid because I felt that I had a problem that I shouldn’t have contacted the healthcare service for, that I should have waited because there are others with bigger problems. *(#9).

*It was hard to be denied care, and not be able to take stand for yourself because I didn’t have the strength to tell them that something was wrong, that I really needed help. When I look back on it, I think that’s actually not okay. *(#18).

Several of the women tried to make telephone contact with the *Breastfeeding clinic* at the university hospital, at the study site, but never received direct contact. Instead, they were supposed to leave a message and then a midwife would call back. The recorded telephone message was not appropriate for women with a possible breast abscess, but they left a message anyway. The difficulties in making contact were experienced by the women as *bad, frustrating, odd*, and *troublesome,* and they became worried. Most participants wanted an easier way to make contact with the service. However, some women thought it was an acceptable way to make contact and felt the breastfeeding centre easily accessible.

#### Assessment and diagnosis of the breast abscess

A range of different healthcare professionals assessed the participants, including physicians without specialist training, gynaecologists, and medical students. They assessed the breast by *looking, squeezing, touching,* and *pulling* which were experienced by the women as *unusual, strange, left out, uncomfortable,* and *objectifying*, as such a private and intimate part of the body was openly exposed and judged. Diagnostic ultrasound was used as part of the assessment process in order to identify the breast abscess and to judge the appropriate time for draining. Sometimes, the physician did not order or carry out any ultrasound examination and did not know the treatment procedure for a breast abscess. A first-time mother said: *It was like he was completely unaware of what he was supposed to do with my condition, it was like it was the first time he saw a breast abscess* (#9).

In some cases, women reported that a physician performed breast ultrasound even though they appeared to be unfamiliar with the machine. Participants also described physicians as having concurrent duties and sometimes had to postpone the breast examination, for example, to perform an emergency caesarean section. The women interpreted clinicians’ behaviour as indicating that caring for mothers with a breast abscess was a lower priority than other conditions.

#### Demanding experience of breast abscess when breastfeeding

Many participants reported a significant reduction in milk production. They developed symptoms of the breast abscess over a prolonged period, which reduced their overall wellbeing. The demanding experience of breast abscess was described as a *hard bullet*, *lump* or *hard plate under the skin*, which at first did not hurt and did not disappear after feeding. The breast abscess could also be described as a *cannon ball*, *a large pebble* in the breast, or *like a golf ball* raised above the skin of the breast.

The area around the abscess was experienced as *hot, tender* and *reddened*, and the colour of the skin around the abscess was described as *red, blue* or *transparent in a yellowish tone*. The women described that the skin became *glossy, beginning to crack or flake*, and when the abscess became mature, they felt that the breast would burst. Some women described the area over a superficial newly developed abscess as *spongy, soft,* and *sunken with a clear ring around it*.

The pain associated with the breast abscess was described as varying between no pain and terrible pain. The pain often escalated in connection with the onset of fever and when pus re-accumulated within the abscess after drainage. Often the pain increased as the abscess grew and sometimes painkillers were needed to be able to handle the pain.

The infected breast was *swollen, tense,* and gave a *throbbing sensation*. Some women found it difficult to lift their arm and they were reluctant to touch the breast or have clothes against it. It was stressful not to be able to be able to be close to their child and that had a negative impact on the women’s mental wellbeing.

#### A long, protracted waiting time to undergo drainage of the breast abscess

The women were asked by their healthcare professionals to observe the abscess and advised to make prompt contact with the health care service when the abscess became *mature*. Often the abscess matured slowly and they had a protracted wait until they could undergo drainage of the abscess, which they experienced as a *difficult, tough, hard,* and as *tiring process*, making them feel depressed and neglected. A first-time mother described this as, *I felt very sorry for myself. I thought it has to be enough. The whole period of time and having an infection made me very depressed* (#15).

The women became worried when they did not know how long they would have to endure until the time was right for drainage and they described a number of fears: not becoming better, their breast being deformed, the need for an emergency operation for a ruptured abscess, or even the need for removal of their breast. The women lacked knowledge for how long time it could take from a mature abscess until rupture occurred. One multiparous woman experienced a breast abscess rupture and described as, *the breast exploded and I had 'raspberry sauce' all over my sweater* (#6).

#### Mixed experience of the breast abscess treatment

The breast abscess was treated in various ways: by drainage with Pig-tail or incision with placement of a drainage tube, and/or needle aspiration (Table 4). Local practice is to use a pig-tail drain for collections where the fluid cavity is > 3 cm [19]. The fear of discomfort and pain was the hardest thing for the women. Mostly the women received local anaesthesia before the breast abscess treatment procedure, either by infiltration, cream, gel or spray. Women experienced the procedure as *good, acceptable*, *a small nip* or with *no worries*. But, for others, significant pain was still experienced and they asked for more and sometimes received additional anaesthesia. One multiparous woman said:

*He then just forced that* [needle] *in my tit without anaesthesia, the pain was insane, the worst I had experienced, like torture, he just went on, didn’t give a shit how painful it was for me, I became so shocked over the whole thing, I will never forget that.* (#2).

A few women reported unsuccessful and painful attempts at placing a Pig-tail drainage. The physicians had different attitudes about when a breast abscess should be drained, which was perceived by the women as unprofessional. One first-time mother described: *‘Why do they give different advice?’, I felt unsecure, ‘Who am I supposed to listen to?’* (#1).

The women were nervous during the Pig-tail drainage of the breast abscess, and the procedure was experienced both positively and negatively. A positive experience was evoked when a nurse was holding their hand when the healthcare professionals described the procedure in detail and had an understanding of the pain involved in the procedure. One multiparous woman said: *I had trust in them when they knew what to do, were skilled, and in the same time human and treated me kindly* (#7).

Other women described the Pig-tail drain insertion as *scary, painful, nasty, unpleasant,* and *frightening*, and that the physician was insensitive to the women’s experience. One primiparous woman described that it was the first time the physician had drained a breast abscess by Pig-tail and that she did not like *to be a guinea pig* (#14). Many women reported that breastfeeding was difficult during the treatment period; some had feelings of *disgust, frustration*, and *distress*, particularly with the drainage tube. One primiparous woman described: *I didn’t want to touch that drain tube, I thought it was disgusting that there was something artificial inside my body* (#1).

The women had to flush the Pig-tail drain themselves which was experienced at first as *dodgy, nasty,* and *scary*. One multiparous woman said, *It was scary at first..., but I quickly adjusted to it* (#7).

The Pig-tail drainage helped to reduce the tenderness and tension in the breast. Participants understood that the condition would eventually be better. They appreciated being able to flush the Pig-tail drain at home instead of going to the hospital for the procedure. The flushing was experienced as a sensation of *tension* and *pulling* inside the breast. After the procedure, women felt relieved when the abscess cavity was empty and the pressure/tension had diminished. Some women would have preferred more help from the healthcare professionals in the procedure of flushing. When the amount of pus decreased the Pig-tail drainage were removed. The removal procedure was experienced as *insane* (#1), but could also be experienced as *smoothly* and *not at all dangerous* (#7).

In some cases, the abscess refilled with pus, and the women were advised by the physicians to squeeze the breast in order to empty the abscess cavity. A first-time mother described this procedure, after incision under general anaesthetic for placement of a drainage tube as:

*I took a shower; it was just pouring out pus, for sure half a decilitre, without any exaggeration, it was completely grotesque, it’s hard to believe that the body is able to produce such amounts, ‘Where does it come from?’.* (#15).

Eight women were managed by needle aspiration of the breast abscess. One of the multiparous women reported that the physician did not know which needle should be selected for the procedure; *I was terrified when he said that he didn’t know what kind of needle he was going to choose, he put 12 needles on a row at a table, a cold sweat broke out on me. It was completely unrealistic, like a horror film scenario* (#2).

After the needle aspiration, one first-time mother felt ignored and did not know how to take care of herself, she said: *I have not received any information from the staff on how to take care of it* [the wound]*; that information should have been given to me at the breast centre* (#11).

Most participants continued breastfeeding during their breast abscess treatment. When the breast abscess was located in the upper part of the breast, it did not hinder attachment of the baby to the breast. Localised breast pain was experienced by some women, and was aggravated by the baby suckling and touching the breast with their hands while feeding, and explained as: *It was a bit tricky, because every time I broke down when I had to put her on that breast. I thought it’s going to hurt, so I kind of tried to mentally prepare myself every time* (#10).

For some women, breastfeeding was also difficult because their baby did not want to attach due to the abscess mass, or to the changed shape of the nipple/areola/breast and yet their healthcare professionals were pressuring them to continue breastfeeding. This emotionally charged situation led to feelings of panic and anxiety.

The majority of women were determined to continue breastfeeding; they wanted to persist despite all of the difficulties experienced. Two first-time mothers explained: *I had decided that ‘I am really going to breastfeed’. I actually didn’t give up, it wasn’t on my agenda to quit, I was just going to fight for it somehow* (#1). *I managed to breastfeed for 6 months, which was my first goal ... but I’m probably most proud and satisfied that I didn’t give up* (#16).

Two of the women did not breastfeed during the breast abscess treatment. One woman with three children restarted breastfeeding after the treatment (#7); while a primiparous woman reported that her baby did not want to breastfeed even with a nipple shield so she had to formula feed instead. She said: *Yes, I had pretty much accepted that it’s not worth holding on and continuing to fight* [to maintain breastfeeding] *when you understood that she didn’t want to* (#4). The other women continued to breastfeed during the treatment, but a few eventually ceased because of ongoing pain and being unable to hold their baby, or advice from their physician that cessation was necessary to resolve the abscess. Some women found their milk production was severely affected by the abscess, and they need to stop breastfeeding because their baby was not satisfied. Two primiparous women explained:

*I was stressed. I sat there with the damn breast pump, and felt like ‘a fucking cow’ and nothing came out, well ugh, it wasn’t fun* (#18).

*That breast was so ‘emotional’, so I felt that I didn’t want to deal with that breast ... the pus poured out … I knew this won’t go, so my focus was that she* [the baby] *would be happy, that was most important* (#15).

#### Treatment follow-up

After treatment for the breast abscess, women were offered a follow-up appointment at the hospital’s *Breastfeeding clinic* and/or at *Breast centre* when it was required. At the follow-up visit at the *Breastfeeding clinic*, the breast was examined by ultrasound in order to assess resolution of the abscess. The follow-up visit, was mostly experienced by the women as good, and they described the healthcare professionals as friendly and competent which made them feel *satisfied* and *secure*. One first-time mother treated with Pig-tail drain said, *Great that they checked with ultrasound…, that they did it properly, then I knew it was over for real, that it is not only my responsibility if anything was not alright* (#4)*.* One multiparous woman treated with needle aspiration regarded the check-up as unnecessary because she felt there was nothing left of the abscess, *I just had it confirmed that it was all right* (# 10).

Some women could still feel a lump after their treatment and were worried that pus might still be present in the abscess cavity. Sometimes a needle aspiration was carried out during the follow-up and one first-time mother described this as: *I could see on the ultrasound that the size of the lump decreased ... it became clear that it was breastmilk* (# 16).

Another first-time mother was worried about still having a lump in the breast and explained, *The staff told me that it could take several months for the lump to totally disappear. They looked with ultrasound and I felt good that they checked that nothing was wrong* (#3).

Some women received a follow-up visit at the hospital’s *Breast centre* for cancer screening or assessment for cosmetic treatment due to the post-treatment scar. These women would have preferred feedback of this check-up by the physician who had treated them at the *Breastfeeding clinic*. One first-time mother explained, *I would have liked if they had told me that they had seen the examination outcome and then said to me: ‘This is completely normal after a breast abscess, it has healed nicely and everything is fine’; but I was wondering: ‘Who is responsible?’* (#14).

### Theme 2: importance of adequate professional care

The second theme ‘Importance of adequate professional care’ incorporated four sub-themes, and are presented in Table 2.

#### Continuity of care preference

During the process of breast abscess treatment, women received care from several physicians. Some women were not bothered about the lack of continuity because the group of healthcare professionals were experienced as *nice, professional,* and *competent* which led to safety. One multiparous woman said, *It was about the atmosphere at the breastfeeding centre, it was like a ‘big family’, they stick together even if they didn’t have one regular physician* (#10).

Without continuity of care, the women had to explain and repeat their story several times. The women became uncertain if everything had been noted in their medical record or if the physicians not had read what had been documented. Two first-time mothers said: *It’s important that everything is written in my medical record, so I don’t need to keep track of the procedure* (#14); and *I had to repeat myself, I didn’t know if I left something out, ‘Is it my responsibility?’* (#8).

The kind of treatment and information often changed between each physician. Then the women felt left out, lacking trust and questioning the physicians’ competence, they had to speak for themselves and find knowledge about the process of treatment elsewhere.

If the system provided continuity of care, a physician could have the primary treatment responsibility. Women felt the number of hospital visits and treatment procedures could have been reduced. If women were well known by the healthcare professionals:

*...then there is less that can go wrong, if there are several* [physicians] *and all make their own assessment..., then something easily can go missing or been understood wrongly. *(#18)

*...then they probably not had forced a needle in my breast so many times and failed. Now it was like different physicians thought that they could take a chance, if so, they maybe had done it once, but not three times! It really hurt after each time they failed. *(#14).

Some of the women did experience continuity of care and they described feelings of trust and safety; that the healthcare professionals were in control, and they did not have to repeat their story. The women regarded continuity of care as important because breastfeeding is a delicate issue and the breast a sensitive area of the body.

#### A wish for respectful communication and information sharing

Respectful communication by the healthcare professionals were important for the women. When the healthcare professionals took time to share information, explain the process of treatment, and describe what they were doing, the women understood and were satisfied with the care given despite an unpleasant treatment, they felt *secure, comfortable,* and *supported*.

The women appreciated consistent and repeated oral and written information in detail. They also appreciated seeing pictures of a mature breast abscess. Likewise, when simple questions were asked and when the healthcare professionals had a calm and soft voice were valued. A multiparous woman, treated by Pig-tail, described her experience as, *they* [the staff] *explained all steps in a good way, I didn’t need to go out on the Internet Googling, I could mentally prepare and not be shocked or sad during the procedure at the hospital* (#7).

When the communication and information sharing were deficient, the women described the healthcare professionals as *harsh* and *unsympathetic*, without taking their feelings into account. The women would have appreciated if they were given information in words they understood about the treatment given and how they were supposed to take care of the wound. When this was lacking some women did not know how to prepare for the treatment procedure, experienced the situation more serious than it was, felt rushed, and that they did not want to be alone with the physician. Two women explained:

*I said Ow!, It hurt when he forced the needle in my breast and he just said ‘Don’t complain’, it* [the pain] *will soon be gone!’, probably he was right, but he could said it differently, he could have said ‘I understand it’s painful, but now we will do this, Is that okay with you?’. *(#8).

*‘Oh, this is not too bad!’, I became sad, she was harsh, I needed help and support in that situation, I became sad that it was another breast abscess, and she said; ‘Why are you sad?’ and, ‘This is nothing to be sad about’. Because of that, I didn’t want to see her again. *(#6).

It was important for women to be able to ask questions about the breast abscess and its treatment, but that was not always offered to them. Then, they became worried about what was going to happen with them, and if the breast abscess, were going to have an impact on future breastfeeding. When the healthcare professionals talked to each other helped each other and asked each other for advice the women experienced this as good and positive, they had faith in them and felt *in good hands,* and became relaxed.

#### The importance of professionalism

The healthcare professionals were regarded as competent when they knew how to treat the breast abscess, had previous experience of this kind of treatment, and had an understanding and respect for the women’s experiences. When the healthcare professionals had these qualities the women felt *safe, grateful,* and *well looked after*. One multiparous woman said, *I have met friendly staff with competence, I felt secure and they have helped me to move on, even if it has been mentally demanding* (#10).

The physicians were regarded as incompetent when they did not know how to treat the breast abscess, and when the women were not treated correctly, or did not receive understanding and respect. Examples, of incompetence described by the women were when no anaesthesia was given, when the physicians argued with each other if antibiotic treatment was necessary or when the right time for drainage was, and if it was the first time for them to perform the procedure. The women then felt *shocked, vulnerable, uncomfortable, dissatisfied* and as *being a guinea pig* for the physician. They felt their care was contradictory and they had to be the expert themselves. Two first-time mothers explained this:

*They* [the staff] *had different views when it was the right time for drainage, I was present during their conversation, there were many different strategies. *(#13).

*I should not have been the one with the right knowledge; you have the trust that the health care should know how to treat it, so it went very bad; I had my infection for a very long time. *(#15).

At the hospital-based *Breastfeeding clinic*, the women were cared both by physicians and by midwives. The women describe the staff being in control and had the correct documentation in the medical records. The healthcare professionals were experienced as *sympathetic, nice, friendly, caring, attentive, welcoming, calm,* and *respectful*. Because of this behaviour, the women felt *supported, safe*, and that their worries and fears were acknowledged. One multiparous woman said:

*They have done more than asked for, in helping me... I have cried in so many hugs, it’s like coming home, it’s like having a mother taking care of me, so fantastic, I needed it, that was proper support. *(#6).

#### Role of caregiver’s gender in the care given

Some women would have preferred to have the right to choose a female physician if desired. When the physician was female, the participants thought that they were able to develop a deeper understanding for what they had gone through in regards to childbirth and breastfeeding. When a male physician cared for them, some women described the care as *objectifying, strange* and being *uncomfortable*. The women also said that the gender issue should not be generalized and it is more important to be treated with respect and integrity regardless of gender. One multiparous woman stated:

*It’s not possible for a man to know what it’s like to have breasts, she* [the physician] *was a woman, she also had breasts, we talked about breastfeeding, she had children, and for me it’s very important that it was a woman who had breastfed, who understood my struggle for my child’s survival, how much I love my child and do everything for my child. She was a fellow human being, she was there for me which meant a lot, I am very grateful that I got to meet her after that horrible experience. *(#2).

## Discussion

This study provides insight into the difficulties women experienced while negotiating treatment for a breast abscess. Initially, they were uncertain where to ask for professional help and then they had a long wait for *the right time* to undergo the unpleasant and painful treatment of their breast abscess. We found that women felt it was important to receive professional care with respectful communication, continuity of care, and to receive adequate information. Unfortunately, they did not always receive this care. Women with puerperal breast abscesses often slip between medical speciality areas. No longer under the care of obstetricians and by maternity services, their problem is too complicated for general practitioners or emergency departments, but not regarded as serious by breast surgeons.

The women in our study believed that the system of booking an appointment for assessment of their breast abscess should have been easier. Since it is paramount that breast infection is diagnosed and treated early [10], women with a possible breast abscess need to be referred without delay to the appropriate health professional [37]. All healthcare professionals should have at least a basic understanding of the importance of breastfeeding and where to refer women, if unable to assist in diagnosis and management of breast infections themselves [38]. Women need to feel confident that whomever they turn to for breastfeeding support will be able to provide appropriate assistance [39].

We found that our study participants were not always looked after by a breastfeeding expert, and some healthcare professionals questioned why they had come for care. General practitioners see a high proportion of women in the postnatal period globally, have often little or no formal education about breastfeeding in their training programmes and may not be aware of the evidence available [39]. Breastfeeding knowledge is not explicit stated as a goal in the specialist training in Sweden, but include for example basic surgical skills and ultrasound [40].

Many women in our study experienced a protracted waiting time until their abscess was drained. Clinically it can be difficult to differentiate an abscess from mastitis so diagnostic ultrasound is required. Using ultrasound, an abscess appears as a hypoechoic collection of variable shape and size, often with a thick echogenic periphery where increased vascular flow is identified [21]. A typical management regime is repeated ultrasound until a collection develops or the problem resolves. A multidisciplinary team, preferable including a radiologist, should follow a treatment algorithm, which may require multiple ultrasound scans [21]. Christensen et al. reported 86 (97%) out of 89 patients with puerperal abscesses were treated with one round of ultrasound-guided drainage; and individuals had a mean of four scans (range 1 to 10) including follow-up [41]. It would be helpful for clinicians to inform women that the process of abscess development may take days or weeks and to establish care plans which facilitate management until the problem has been resolved.

Respectful communication by healthcare professionals were important for the participants in our study. The women appreciated when the professionals took time to share information, explained the process of treatment, described what they were doing, and when they were allowed to ask questions. To be able to support women with breastfeeding difficulties, professional communication and clinical skills are required [39]. Healthcare professionals not always give mothers a chance or enough time to ask questions about issues they do not understand concerning childbirth [42]. When feeling uncertain about one’s own health, the experience of the illness itself, the diagnosis, unknown aetiology of symptoms and waiting-time for results can be an emotional burden, leading to stress, anxiety and feelings of vulnerability [7]. Poor communication between professionals and patients can lead to negative outcomes in healthcare such as compromised patient safety, patient dissatisfaction, discontinuity of care, and inefficient use of valuable resources [43, 44]. Furthermore, a lack of a good relationship between women and their healthcare professionals may result in conflicting advice and unsupportive breastfeeding care. When healthcare professionals are non-judgmental, encouraging, reassuring, sympathetic, patient and understanding, women experience them as supportive [45].

When the communication and information sharing were deficient, the women in our study described the healthcare professionals as harsh and unsympathetic, without considering their feelings. If patients are not informed about their treatment such as breast abscess management, due to healthcare professionals not sharing important information, patients may feel ignored about their care [46]. Patients understanding of safety has been defined as healthcare professionals caring for their dignity and well-being. Patient safety has also been portrayed as being loved and respected by healthcare professionals. Furthermore, safety has been associated with a feeling of being in ‘good hands’, having a respectful communication and a good relationship with healthcare professionals, rather than with factual knowledge of potential errors and hazards [46].

Some of the women in our study did experience continuity of care and they described feelings of trust and safety; the healthcare professionals were in control, and they did not have to repeat their story. The women regarded continuity of care as important because breastfeeding is a delicate issue and the breast a sensitive area of the body. Continuity of care is known to facilitate personalised care, development of trust [47], and reduces uncertainty around one’s own health and risk [7, 46]. Women’s experiences of treatment for breastfeeding difficulties like mastitis, the interaction with an empathetic physician and midwife who continuously care for them throughout the hospital stay is appreciated and save women from feeling like a parcel being passed around in a healthcare system [6].

The healthcare professionals were regarded by the women in our study as competent when they knew how to treat the breast abscess, had previous experience of this kind of treatment and had an understanding and respect for the women’s experiences. Characteristics of healthcare professionals that contribute to a sense of trust for patients include confidence in their clinical competence and their personal knowledge of the patient [46]. In order to develop a trustful relationship it is important for professionals to spend enough time with the patient [48, 49], which was not always the case for some of our participants. To provide person-to-person contact with warmth and proximity, and being considerate, is known to reduce patient anxiety [48]. The women in our study described the professionals they saw were not always knowledgeable in the management of puerperal breast abscess. Multidisciplinary efforts to improve clinical breastfeeding practice including physicians, midwives, lactation consultants, paediatricians and registered nurses are preferable [33]. In Sweden, the main caregiver during the antenatal and postnatal time-period for a woman with no medical complications is a midwife, with educational breastfeeding training [24]. When there are breastfeeding difficulties, additional support are available to women by midwives and physicians, some as educated lactation consultants, at 26 Breastfeeding clinics across Sweden [26].

### Strengths and limitations

Our study is one of the first qualitative studies describing women’s puerperal breast abscess experiences. One of the study’s strengths is the lengthy interviews using an interview guide, allowing participants to talk freely. However, it is unknown if face-to-face interviews would have given a more detailed description of the phenomenon under study. The authors’ pre-understanding about puerperal breast abscess management may have influenced the data analysis and should be taken into account in the transferability of the findings. We acknowledge that the study participants were all recruited from one area, and some of their experiences might have been specific for that location. The findings resonated with the authors based in two countries. Qualitative studies do not aim to be generalisable, but to understand people’s experiences. It would be useful to replicate the study in other settings to continue and to deepen our understanding of the phenomena under investigation. Future research could explore healthcare professionals’ experiences of managing women with puerperal breast abscesses.

## Conclusions

We identified many areas of care that women with breast abscesses found lacking. They did not know where to ask for professional help and had an extended wait for the right time to undergo an unpleasant and painful drainage of their breast abscess. Although women felt it was important to receive professional care with respectful communication, continuity of care, and to receive adequate information, their care often fell short. Healthcare professionals urgently need adequate training in order to deal with breastfeeding problems and be able to offer women-centred care.

### Supplementary Information

**Additional file 1.**

## Data Availability

The datasets generated and analysed during the current study are not publicly available due [the ethics committee restricts secondary use of the current data] but are available from the corresponding author on reasonable request.

## Abbreviations

ICD-10: International Statistical Classification of Diseases and Related Health Problems
MRSA: Methicillin-Resistant *Staphylococcus Aureus*
